# CD137-CD137L Interaction Regulates Atherosclerosis via Cyclophilin A in Apolipoprotein E-Deficient Mice

**DOI:** 10.1371/journal.pone.0088563

**Published:** 2014-02-10

**Authors:** Yuefeng Li, Jinchuan Yan, Chao Wu, Zhongqun Wang, Wei Yuan, Dongqing Wang

**Affiliations:** 1 Department of Cardiology, Affiliated Hospital of Jiangsu University, Zhenjiang, China; 2 Department of Radiology, Affiliated Hospital of Jiangsu University, Zhenjiang, China; King's College London School of Medicine, United Kingdom

## Abstract

**Background:**

Our previous studies showed that increased levels of cyclophilin A (CyPA) may be a valuable marker for predicting the severity of acute coronary syndromes and that interruption of CD137-CD137L interactions diminished the formation and progression of atherosclerosis in apolipoprotein E-deficient (ApoE−/−) mice. Here, we sought to determine whether the proinflammatory factor CyPA is involved in atherosclerosis regulated by CD137-CD137L interactions.

**Methods and Results:**

A constrictive collar was placed around the right carotid arteries of ApoE−/− mice that were fed a high-fat diet to induce atherosclerotic plaque formation. After that, the mice were intraperitoneally injected with anti-CD137 or anti-CD137L in the presence or absence of the recombinant lentiviral vectors LVTHM-CyPA or pGC-FU-CyPA, respectively. Interestingly, activation of CD137-CD137L was negatively correlated with CyPA expression in vivo and in vitro. Stimulating CD137-CD137L interaction significantly increased CyPA, which was concurrent with the upregulation of proinflammatory cytokines, chemokines and matrix metalloproteinases and resulted in the promotion of atherosclerosis in ApoE-/- mice. Silencing CyPA could eliminate these effects, and restoration of CyPA effectively and consistently attenuated the atherosclerotic suppression phenotypes elicited by the blockade of CD137-CD137L.

**Conclusion:**

These observations suggest that CD137-CD137L interactions mediated via regulation of CyPA contribute to the progression of atherosclerosis.

## Introduction

Accumulating evidence suggest that inflammatory reactions involving interactions among the blood, vascular and immune cells, including activated T cells and macrophages, are involved in the development of atherosclerosis. Previous studies from our lab as well as others have shown that several members of the tumor necrosis factor (TNF) superfamily, including CD40-CD40L, LIGHT, and OX40-OX40L, are involved in atherosclerosis development [1]–[5]. Recently, data from our laboratory demonstrated that an elevated level of soluble CD137 (sCD137) is associated with increased cardiac risk and may be a marker of plaque instability. Furthermore, inhibition of CD137-CD137L signaling significantly inhibited the formation of atherosclerotic lesions in apolipoprotein E-deficient (ApoE−/−) mice [6]–[8]. Consistent with our data, it has been demonstrated that CD137-CD137L interactions in the vasculature may lead to the progression and increased vulnerability of atherosclerotic lesions via augmented leukocyte recruitment, increased inflammation, and the development of a more disease prone phenotype [1]. However, the underlying mechanisms of CD137-CD137L interactions in the regulation of atherosclerosis still remain largely unclear. In view of this, our objective was to investigate the signaling interaction between CD137-CD137L and atherosclerosis.

CyPA, a 20 kDa chaperone protein of the cyclophilin family, has been suggested to mediate a variety of cardiovascular diseases, including vascular stenosis, atherosclerosis, and abdominal aortic aneurysm (AAA) [9]. Within atherosclerotic plaques, it has been reported that vascular smooth muscle cells (VSMCs), monocytes and endothelial cells (ECs) can secrete large amounts of CyPA stimulated by reactive oxygen species (ROS), which are induced by mechanical stretch; pressure, shear stress; or environmental factors such as hypoxia; and secreted factors, such as angiotensin II (AngII) [10]–[12]. Furthermore, studies have shown that CyPA knockout mice demonstrated a remarkable reduction in atherosclerosis and that CyPA was atherogenic by enhancing LDL uptake, adhesion molecule expression, and inflammatory cell migration, suggesting that CyPA is a potential target for cardiovascular therapies [13].

In this study, we demonstrated that CD137-CD137L interaction contributes to the development of atherosclerosis; CyPA is significantly suppressed by the inhibition of CD137-CD137L and is stimulated by the activation of CD137-CD137L signaling pathway in ApoE−/− mice; CyPA inhibition impairs the proatherogenic role of anti-CD137; and ectopic expression of CyPA diminishes the atherosclerotic suppressor function of anti-CD137L, indicating that the effect of CD137-CD137L on atherosclerosis is at least partially mediated by regulation of CyPA.

## Materials and Methods

### Lentivirus vectors

We used a pGC-FU-CyPA and LVTHM-CyPA plasmid from a lentivirus-based gene library (Open Biosystems, USA), which can be used to overexpress or inhibit CyPA, to package the lentiviruses pGC-FU-CyPA (The sequences of used primers: 5′CGCGGATCCGCGATGGTCAACCCCACCGT-3′, and CCGGAATTCCGGTTAGAGCTGTCCACAG-3′) and LVTHM-CyPA (sequence: TATGGCGTGTAAAGTCACCAC). Plasmid DNA and the transfection complex DNAs were transfected into 293 T cells through the use of Arrest-In reagent, according to the manufacturer's instructions. A validated, non-silencing, scrambled control was used as a negative control. The lentiviruses were collected from the supernatant. The titers of pGC-FU-CyPA and LVTHM-CyPA averaged 1×10^10^ TU/ml and 1×10^10^ TU/ml, respectively.

### Mice

The study protocol was reviewed and approved by the Animal Care and Use Committee of Jiangsu University. 70 male ApoE−/− mice aged 8 weeks were purchased from Vital River Laboratories (Distributor of Jackson Laboratory, Beijing, China) and housed under standard conditions of humidity, room temperature and dark-light cycles with plenty of chow and water. An atherosclerotic plaque model was produced by rapid perivascular carotid artery collar placement in ApoE−/− mice as described previously [14]. After collar-implant surgery, the mice were randomly divided into the following groups: the CD137-CD137L activated group (anti-CD137, n = 10, injected with 200 µg anti-mouse CD137 mAb, eBioscience, USA); the CD137-CD137L inhibited group (anti-CD137L, n = 10, injected with 200 µg anti-mouse CD137L mAb, eBioscience); the negative control group (NC, n = 10, injected with 200 µg isotype antibody, eBioscience); the CD137-CD137L activated group with LVTHM-CyPA or negative control siRNA injection (anti−CD137+LVTHM−CyPA, anti−CD137+LVTHM−NC, n = 10, injected with LVTHM-CyPA or LVTHM-NC 10 µL and anti-CD137 200 µg); the CD137-CD137L inhibited group with ectopic CyPA or the empty vector control treatment (anti−CD137L+pGC−FU−CyPA or anti−CD137L+pGC−FU−NC, n = 10, injected with 200 µg anti-mouse CD137L mAb combined with GC−FU−CyPA or GC−FU−NC, 10 µL ).

### Cell culture

The VSMCs were isolated from the aorta of ApoE−/− mice according to previously reported methods [15]. The cells were maintained in DMEM/F12 with 10% fetal calf serum (Gibco BRL, Grand Island, NY) in a humid atmosphere containing 5% CO_2_ at 37°C. The following incubations were performed: isotype antibody, anti-CD137 or anti-CD137L (10, 20, 30, 40 µg/ml).

### Histological analysis of atherosclerotic plaques

Atherosclerotic plaque sections from the carotid artery were harvested as described previously [8], [16] and were stained with hematoxylin and eosin (H&E), CyPA (1∶500, Santa Cruz, Inc.) or α-smooth muscle actin (α-SMA, 1∶300, Santa Cruz).

### RNA extraction and real-time PCR

Total RNA was extracted from the frozen atherosclerotic carotid samples using Trizol (Invitrogen). The expression of CyPA, VCAM-1, MMP-9, IL-6, TNF-α mRNA were assayed using stem-loop reverse transcription (RT) followed by real-time polymerase chain reaction (PCR). All reagents for stem-loop RT were obtained from Applied Biosystems (Foster City, USA). The relative amount of each gene was normalized to β-actin. The primers used are as follows: CyPA: 5′-CAAGGTCCCAAAGACAGCAGA-3′ and 5′-AAGATGCCAGGACCCGTATGC-3′; VCAM-1: 5′-CTGAATACAAAACGATCGCTCAA-3′ and 5′-GCGTTTAGTGGGCTGTCTATCTG-3′; MMP-9: 5′-CCTGGAACTCACACGACATCTTC-3′ and 5′-TGGAAACTCACACGCCAGAA-3′; IL-6: 5′-GAGGATACCACTCCCAACAGACC-3′ and 5′-AAGTGCATCATCGTTGTTCATACA-3′; TNFα: 5′-ACCGTCAGCCGATTTGCTATCTC-3′ and 5′-ACTTGGGCAGATTGACCTCAGC-3′; β-actin: 5′-TGGAATCCTGTGGCATCCATGAAAC-3′ and 5′-TAAAACGCAGCTCAGTAACAGTCCG-3′. The relative expression level of each sample was measured using the 2-ΔΔCT method as described previously [16]. PCR was performed in triplicate.

### Western blot analysis

Protein samples were extracted using a protein extraction reagent (Pierce, USA). The proteins were then resolved by 10% SDS-PAGE and were transferred to PVDF membranes. Protein levels were normalized to β-actin (Sigma). The membranes were blocked and probed with antibodies against CyPA (1∶400), CD137 or CD137L (1∶500) (Santa Cruz). Band detection via enzyme-linked chemiluminescence was performed according to the manufacturer's protocol (ECL; Pierce Biotechnology Inc., Rockford, USA).

### ELISA

Peripheral venous blood was obtained from ApoE−/− mice in each group before euthanasia. Venous blood was drawn into pyrogenic-free blood collection tubes without any additives (Becton Dickinson). The blood samples were immediately immersed in melting ice and were allowed to clot for 1 h before centrifugation (1500×g and 4°C for 10 min). The serum was stored at −80°C until analysis. The levels of serum sCD137 were detected by a sCD137 ELISA kit from Bender Med-Systems (Campus Vienna Biocenter, Vienna, Austria); serum CyPA, MMP-9, IL-6 and TNF-α were measured by enhanced sandwich ELISA protocols (eBioscience), according to the manufacturer's instructions.

### Statistical analysis

Data were expressed as the mean ± SD from at least three independent experiments (vitro experiments) and compared using *t*-test or ANOVA by SPSS version 12.0 software (SPSS, Chicago, IL, USA). A two-tailed *P*<0.05 was considered statistically significant.

## Results

### CyPA was correlated with the activation of CD137-CD137L signaling pathway

**Figure 1:**
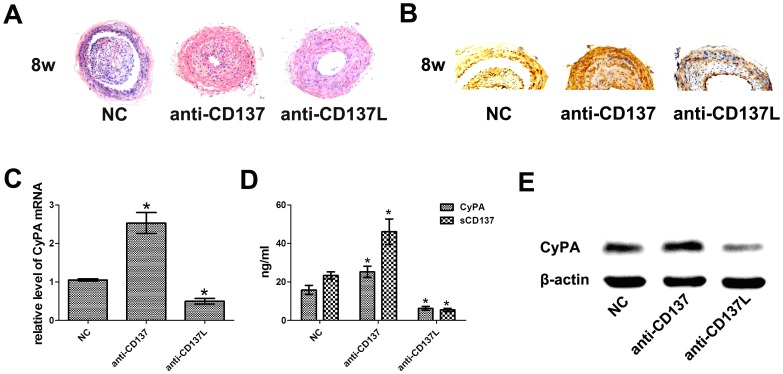
Analysis of plaque areas and CyPA expression in ApoE−/−mice. (A) H&E-stained cross-sections from the carotid arteries in ApoE−/−mice with intraperitoneal injection of anti-CD137 (anti-CD137) or anti-CD137L (anti-CD137L) compared with the control group (NC) for 8-weeks. (B) CyPA expression in plaques obtained from each group detected by immunohistostaining. (C) Serum CyPA concentration and the sCD137 in ApoE−/−mice measured by ELISA. (D) CyPA mRNA in the carotid artery of each group measured by RT-PCR. (E) Western blot analysis of CyPA protein expression, β-actin was detected as the loading control. (*P<0.05)

To study the functional role of CD137-CD137L in atherogenesis, we quantified the plaque area in H&E-stained cross sections of the carotid artery from ApoE−/− mice treated with anti-CD137 or anti-CD137L for 8 weeks. Consistent with our previous study, mice injected with anti-CD137 exhibited significantly less atherosclerosis, whereas a remarkable increase in atherosclerosis was observed in the anti-CD137L group (Fig. 1A). To assess whether CyPA was dysregulated with activation or repression of the CD137-CD137L system, we analyzed the serum concentrations of CyPA and sCD137 in ApoE−/− mice. Increased sCD137L was associated with the upregulation of CyPA, whereas decreased sCD137L was associated with the downregulation of CyPA (Fig. 1C), indicating a regulatory relationship between CyPA and the CD137-CD137L signaling pathway. To strengthen this important finding, we detected the mRNA and protein expression levels of CyPA in plaques obtained from ApoE−/− mice. CyPA was expressed positively but weakly in the anti-CD137L group and strongly and positively in the anti-CD137 group compared with the control group (Fig. 1B). Moreover, the results of RT-PCR and Western blot analyses displayed similar tendencies (Fig. 1D, E). These observations suggested that CD137-CD137L signaling is a significant, atherosclerosis-promoting factor and is strongly associated with the expression of CyPA. These findings raise the possibility that CyPA may play a role in CD137-CD137L interaction-induced atherosclerosis development and encouraged us to further evaluate the role of CyPA in the CD137-CD137L system.

### CD137-CD137L signaling is involved in the regulation of atherosclerosis via CyPA

**Figure 2:**
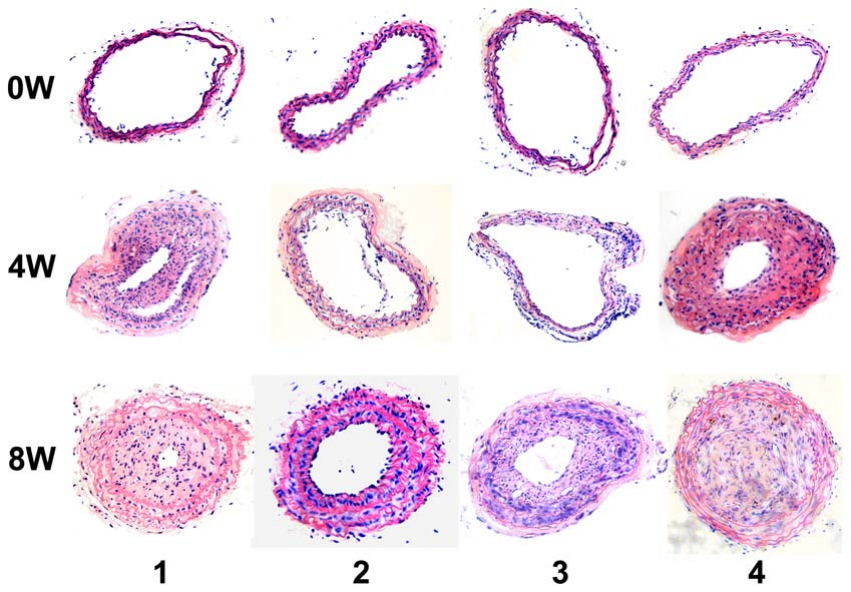
CD137-CD137L signaling involved in the progression of atherosclerosis. H&E-stained cross-sections from the carotid arteries 4 weeks (4 W) and 8 weeks (8 W) after collar placement in ApoE−/−mice intraperitoneally injected twice weekly with anti-CD137 or anti-CD137L in the presence or absence of LVTHM-CyPA or GC-FU-CyPA, respectively. (1. anti−CD137+LVTHM−NC, 2. anti−CD137+LVTHM−CyPA, 3. anti−CD137L+pGC−FU−NC, 4. anti−CD137L+ pGC−FU−CyPA)

**Figure 3:**
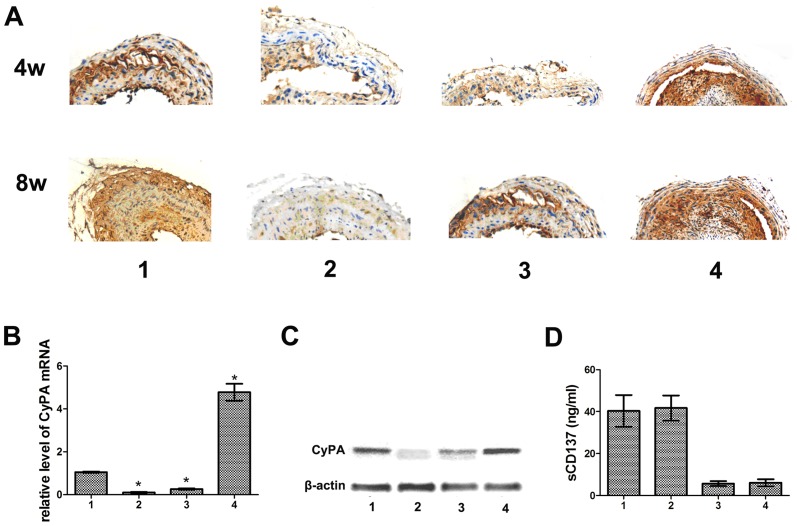
CD137-CD137L interaction regulated atherosclerosis via CyPA. (A) Representative photographs of immunohistostainings against CyPA in plaques obtained from each group. (B) RT-PCR analysis for CyPA mRNA expression level. (C) Protein levels of CyPA were analyzed by Western blot. (D) sCD137 levels in ApoE−/−mice from each group detected by ELISA. (1. anti−CD137+LVTHM−NC, 2. anti−CD137+LVTHM−CyPA, 3. anti−CD137L+pGC−FU−NC, 4. anti−CD137L+ pGC−FU−CyPA) (*P<0.05)

Given the aforementioned findings, we intended to explore the role of CyPA in atherosclerotic lesion formation regulated by CD137-CD137L interaction. Mice were treated with anti-CD137 or anti-CD137L followed by CyPA knock down or CyPA restoration, respectively, and their carotid arteries were evaluated by H&E staining. Four weeks after collar insertion, intimal thickening was detectable in the area of the collar. Eight weeks after collar insertion, a significant increase in intimal surface area was present in each group. Fewer plaques and thinner intimal surface areas were observed in the anti-CD137+LVTHM-CyPA group compared with the anti-CD137 group. Whereas the anti-CD137L+pGC−FU−CyPA group displayed significantly increased cap collagen content and thickened fibrous cap with increasing fibrous tissue on histological examination compared with the anti−CD137L+pGC−FU−NC group, the mice almost completely lost the atherosclerosis suppression that is normally exhibited by anti-CD137L (Fig. 2). Next, to ensure the validity of these results, we examined the expression of CyPA in atherosclerotic plaques from the ApoE−/− mice of each group using RT-PCR, immunohistochemistry and Western blot analyses. As shown in Fig. 3A, B, and C, the mRNA and protein levels of CyPA were increased by anti-CD137 and attenuated by anti-CD137L treatment. CyPA expression induced by anti-CD137 was significantly decreased in response to LVTHM-CyPA injection. Consistently, the restoration of CyPA in the anti-CD137L group effectively and consistently rescued the downregulation of CyPA mediated by anti-CD137L. These data strongly supported our hypothesis that CD137-CD137L contributes to atherosclerosis associated with CyPA. Further, we evaluated sCD137 expression in response to CyPA alteration to investigate the role of CyPA on the CD137-CD137L system. However, no reproducible differences of sCD137 expression were observed between the CyPA knock down or enhanced groups and their related negative control groups (Fig. 3D).

### CD137-CD137L signaling enhanced the production of proatherosclerosis molecules through CyPA

**Figure 4:**
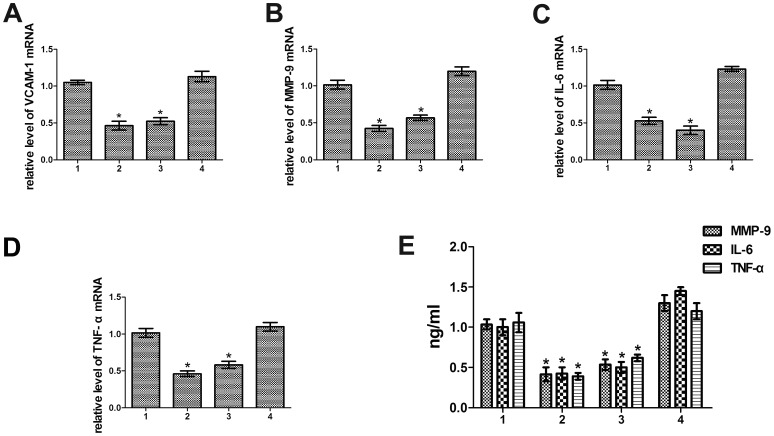
Regulation of proatherosclerosis factors by CD137-CD137L signaling required CyPA. (A-D) RT-PCR analysis for VCAM-1, MMP-9, IL-6, TNF-α mRNA levels in plaques, respectively. (E) Serum concentration of MMP-9, IL-6, TNF-α detected by ELISA. (1. anti−CD137+LVTHM−NC, 2. anti−CD137+LVTHM−CyPA, 3. anti−CD137L+pGC−FU−NC, 4. anti−CD137L+ pGC−FU−CyPA) (*P<0.05)

Previous studies have demonstrated that a remarkable reduction in atherosclerosis in CyPA-deficient mice might be caused by decreased vascular cell adhesion molecule 1 (VCAM-1) expression, which is highly expressed in activated ECs and promoted atherosclerosis [13]. Data from our lab illustrated CyPA treatment altered the production of the critical proatherosclerosis factors MMP-9, IL-6 and TNF-a. To gain insight into whether the downstream effectors of CyPA were targeted by CD137-CD137L interaction, we evaluated the mRNA and protein levels of VCAM-1, MMP-9, IL-6 and TNF-α by RT-PCR (Fig. 4A, B, C, D) and ELISA (Fig. 4E), respectively. As anticipated, the expression of VCAM-1, MMP-9, IL-6 and TNF-α was significantly increased or decreased when treated with anti-CD137 or anti-CD137L, respectively. Forced expression of CyPA in anti-CD137L group restored the expression of these proatherosclerosis factors, while CyPA knock down robustly reversed the increased expression of the proatherosclerosis factors induced by anti-CD137 at both transcriptional and protein levels. In summary, the data presented here exhibited that CD137-CD137L interaction promotes the production of proinflammatory cytokines, chemokines and matrix metalloproteinases, partially through regulation of CyPA.

### The effect of CD137-CD137L interaction on VSMC

**Figure 5:**
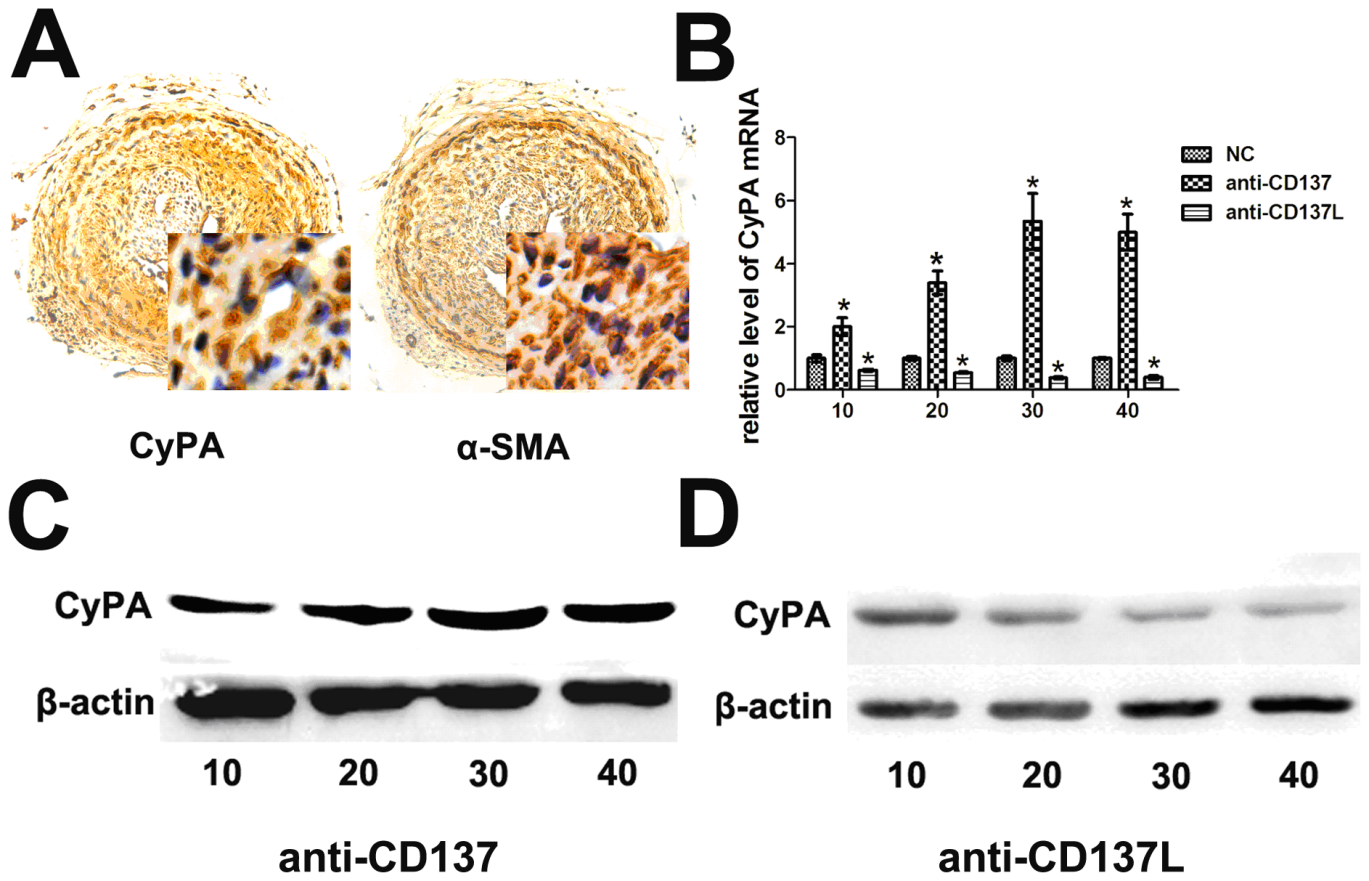
CyPA responded to CD137-CD137L system mainly expressed on VSMCs. (A) Representative histological analysis of the atherosclerotic lesion sites stained with CyPA and α-SMA. (B) The relative mRNA levels of CyPA in VSMCs incubated with anti-CD137 (anti-CD137), anti-CD137L (anti-CD137L) or isotype antibody (NC). (C) Protein levels of CyPA treated with various concentrations of anti-CD137. (D) Protein levels of CyPA treated with various concentrations of anti-CD137L. (*P<0.05).

To confirm the role of CyPA in plaque formation, we observed the distribution of its expression in carotid arteries from ApoE−/− mice. As shown, atherogenic plaques contained a marked increase in the number of VSMCs, as evident from the expression of α-SMA marker in the affected sites. Intriguingly, the distribution of VSMCs was closely overlapped with the areas of highest CyPA expression, especially in mice with enhanced CyPA (Fig. 5A), and CyPA expression was elevated, especially in VSMCs. Next, we wanted to evaluate the functional importance of CD137-CD137L in vitro. Cultured VSMCs were exposed to functional anti-CD137 or anti-CD137L, and the expression of CD137, CD137L, CyPA was characterized. As showed in (Fig S1), CD137 was stimulated with anti-CD137 treatment, while no significant changes of CD137L was observed in both anti-CD137 and anti-CD137L treated group, together with the data form vivo experiments (anti-CD137 activation the well-known downstream genes of CD137-CD137L signaling, while anti-CD137L downregulatied them), all these suggested that anti-CD137 stimulated CD137 expression and anti-CD137L block the interactions between CD137 and CD137L effectively, resulted in stimulation or blocking of the CD137-CD137L signaling respectively. CyPA mRNA and protein levels in VSMCs were induced by anti-CD137 in a dose-dependent manner, reaching a maximum at 30 µg/ml, and were decreased by anti-CD137L, with the maximal effect also occurring at 30 µg/ml (Fig. 5B, C, D).

## Discussion

In atherosclerosis, inflammation underlies the lesion evolution at all stages, from establishment to plaque rupture and thrombosis. Activation of T cells, the central orchestrators of the adaptive immune response, is critically dependent on co-stimulatory signals, which are received through, among others, molecules of the TNF superfamily [17]. CD137 (4-1BB), a receptor in the TNF superfamily, is recognized as an independent co-stimulatory molecule of T cells and activator of monocytes [1], [18]. It is suggested that the CD137-CD137L system is involved in coronary artery disease progression and plaque destabilization. Studies have demonstrated a crucial role of CD137 in multiple stages of atherosclerosis and acute coronary syndrome (ACS) [1], [8]. We recently demonstrated that patients with ACS show increased expression of soluble and membrane-bound CD137 [6] and a significant correlation between CD137 levels and complex coronary stenoses [7]. We also showed that CD137-CD137L interactions could regulate the expression of NFATc1 in ApoE−/−mice in vivo and in vitro [8]. In the present study, in addition to NFATc1, another proatherosclerosis molecule, CyPA, was also found to be regulated by CD137-CD137L interactions. Serum CyPA concentrations were consistently correlated with levels of sCD137 in ApoE−/−mice. The expression of CyPA was positively correlated with the activation of the CD137-CD137L signaling pathway in plaques. We propose that CD137-CD137L interactions are involved in atherosclerotic lesion formation through regulation of CyPA.

CyPA plays an important role in several stages of atherosclerosis. CyPA-mediated VCAM-1 expression appeared to precede lesion formation, suggesting the involvement of CyPA in the initiation of atherosclerotic lesions. During fatty streak formation, CyPA stimulated lipid uptake via its effect on scavenger receptors. In all stages, CyPA regulated inflammation by promoting monocyte adhesion and recruitment as well as contributing to an oxidative environment. The data showed that CyPA was secreted from foam cells, revealing an important role of CyPA in the later stages of atherosclerosis. CyPA-mediated activation of matrix metalloproteinase indicated that CyPA may also play a role in plaque rupture [9], [11]–[13]. Inhibition of CyPA undoubtedly would be a novel therapeutic approach for the prevention of atherosclerosis.

Given the crucial role of CyPA in atherosclerosis and the association between CyPA and CD137-CD137L signaling, we supposed that CD137-CD137L might act on the regulation of atherosclerosis through CyPA. We then tested whether the alteration of CyPA in the CD137-CD137L signaling pathway played a role in facilitating atherogenic plaque formation and progression. The results showed that knock down of CyPA effectively decreased the anti-CD137-induced CyPA and diminished the proatherogenic effect of anti-CD137, whereas restoration of CyPA rescued the anti-CD137L-decreased CyPA and almost completely attenuated the arrested progression of atherosclerosis by anti-CD137L. Further, the co-localization of CyPA and α-SMA staining at atherosclerotic lesion sites suggested CD137-CD137L interaction derived CyPA was especially elevated in VSMCs. This interpretation was further supported by the in vitro data. Moreover, in agreement with previous studies, we displayed that both CD137-CD137L blockade and CyPA inhibition restrained the downstream effectors of CyPA, VCAM-1, IL-6, TNF-α, and MMP-9, and this effect could be reversed by CyPA restoration. The analysis of CyPA downstream effectors further supported an essential role for CyPA in CD137-CD137L interaction and atherosclerosis.

In conclusion, our findings showed that CD137-CD137L interactions could regulate the expression of CyPA in ApoE−/− mice, which can affect the formation of atherosclerotic plaque. From the above studies, it is intriguing to suggest that specific and early intervention directed to the interaction of CD137-CD137L and the blockade of CyPA activation signaling might be potential therapeutic targets for inhibiting the progression of atherosclerotic plaques and subsequently for preventing cardiovascular events.

## Supporting Information

Figure S1 The expression of CD137 and CD137L protein in VSMCs incubated with nothing (1), isotype antibody (2), anti−CD137(3) or anti−CD137L (4) detected by western bolt. (1. VSMCs, 2. VSMCs+NC, 3. VSMCs+anti−CD137, 4. VSMCs+anti−CD137L).
(DOC)
