# Supplementary material for: CD137-CD137L Interaction Regulates Atherosclerosis via Cyclophilin A in Apolipoprotein E-Deficient Mice
Source: PLoS One. 2014 Feb 10;9(2):e88563. doi: 10.1371/journal.pone.0088563 (PMC3919780; doi:10.1371/journal.pone.0088563)
Supplement: Figure S1 — The expression of CD137 and CD137L protein in VSMCs incubated with nothing (1), isotype antibody (2), anti−CD137(3) or anti−CD137L (4) detected by western bolt. (1. VSMCs, 2. VSMCs+NC, 3. VSMCs+anti−CD137, 4. VSMCs+anti−CD137L). (DOC) [file pone.0088563.s001.doc]

**Figure S1**

**
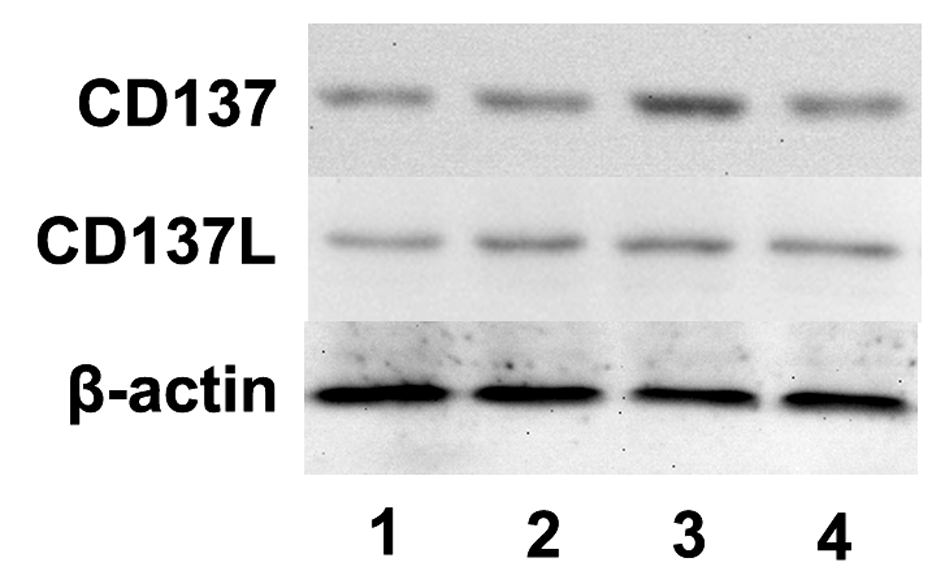
**

The expression of CD137 and CD137L protein in VSMCs incubated with nothing (1), isotype antibody (2), anti-CD137(3) or anti-CD137L (4) detected by western bolt. (1. VSMCs, 2. VSMCs+NC, 3. VSMCs+anti-CD137, 4. VSMCs+anti-CD137L)**.**
